# The dual hypothesis of homeostatic body weight regulation, including gravity-dependent and leptin-dependent actions

**DOI:** 10.1098/rstb.2022.0219

**Published:** 2023-10-23

**Authors:** John-Olov Jansson, Frederik Anesten, Daniel Hägg, Jovana Zlatkovic, Suzanne L. Dickson, Per-Anders Jansson, Erik Schéle, Jakob Bellman, Claes Ohlsson

**Affiliations:** Institute of Neuroscience and Physiology, The Sahlgrenska Academy at the University of Gothenburg, S-41390 Göteborg, Västra Götaland, Sweden

**Keywords:** gravitostat, body weight sensing, leptin, loading, body weight homeostasis, obesity

## Abstract

Body weight is tightly regulated when outside the normal range. It has been proposed that there are individual-specific lower and upper intervention points for when the homeostatic regulation of body weight is initiated. The nature of the homeostatic mechanisms regulating body weight at the lower and upper ends of the body weight spectrum might differ. Previous studies demonstrate that leptin is the main regulator of body weight at the lower end of the body weight spectrum. We have proposed that land-living animals use gravity to regulate their body weight. We named this homeostatic system the *gravitostat* and proposed that there are two components of the gravitostat. First, an obvious mechanism involves increased energy consumption in relation to body weight when working against gravity on land. In addition, we propose that there exists a component, involving sensing of the body weight by osteocytes in the weight-bearing bones, resulting in a feedback regulation of energy metabolism and body weight. The gravity-dependent homeostatic regulation is mainly active in obese mice. We, herein, propose the *dual hypothesis of body weight regulation*, including gravity-dependent actions (= gravitostat) at the upper end and leptin-dependent actions at the lower end of the body weight spectrum.

This article is part of a discussion meeting issue ‘Causes of obesity: theories, conjectures and evidence (Part II)’.

## 1. Introduction

Because of the obesity epidemic and the severe ailments associated with obesity it is of crucial importance to find new treatments for this disease. This could be facilitated by investigating the physiological regulation of body weight. Speakman has proposed that there are individual-specific lower and upper intervention points for the regulation of body weight. According to the hypothesis of Speakman, homeostatic counter regulatory mechanisms are only initiated when body weight passes below the lower intervention point or above the upper intervention point [1,2]. The nature of the main homeostatic mechanisms regulating body weight at the lower and upper ends of the body weight spectrum might differ. Proposed mechanisms for these homeostatic regulations will be discussed in the present article.

## 2. Leptin and body weight biology

**Figure 1.**
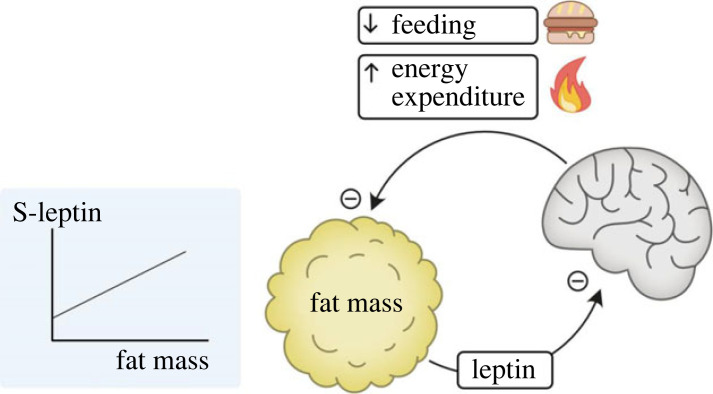
Schematic illustration of leptin's effects in body weight homeostasis. Leptin, produced by white adipose tissue in proportion to fat mass, acts within the brain, especially the hypothalamus, to decrease feeding and increase energy expenditure, as part of a homeostatic feed-back loop to suppress fat mass.

The concept that achieving a stable internal milieu is of importance for life was first discussed by Bernard [3], and later the term ‘homeostasis’ was coined by Walter Cannon to describe this phenomenon [4]. Several parameters in the body were later found to be regulated by homeostasis, such as blood glucose and core body temperature. In line with this general concept, Kennedy proposed a lipostatic model of body fat regulation in 1953 [5], but it was not until 1994 that Friedman and co-workers discovered leptin by using positional cloning in severely obese *ob*/*ob* mice [6], based on the hypothesis put forward by Coleman [7,8]. The leptin system was found to fulfil many of the criteria for being a homeostatic regulator of fat mass [6,9]. Leptin is a hormone produced in fat tissue and then released to the bloodstream in relation to fat mass to exert anti-obesity effects (figure 1). Animals and humans that lack leptin become very obese, with severely increased food intake and also decreased energy expenditure [6,9]. Leptin treatment can rescue this phenotype, indicating that leptin indeed is an important factor for the regulation of body fat mass [6,9,10]. It is assumed that the anti-obesity effect of leptin is mainly exerted via the brain, especially the hypothalamus (see figure 1) [11,12]. Unfortunately, it seems that systemic leptin treatment *per se* is not very effective in treating common obesity [13]. However, this does not exclude the possibility that leptin in combination with a yet unknown cofactor might have the capacity to regulate body weight in common obesity. The lack of effect by leptin treatment in common obesity has been called leptin resistance and is not fully understood, although a number of possible mechanisms have been suggested [7,14,15]. Despite the leptin resistance phenomenon described above, the presence of leptin is crucial in individuals with different grades of moderate obesity, where leptin contributes to avoidance of severe obesity. This is shown by the dramatic obesity in individuals (mice and humans) lacking leptin [9,10].

## 3. Gravity-dependent regulation of body weight

Leptin seems to be the main homeostatic regulator of body weight at the lower end of the body weight spectrum, while the nature of the homeostatic regulator at the upper end of the body weight spectrum is unclear [13,16]. It is possible that the nature of the homeostatic mechanisms regulating body weight at the upper end of the body weight spectrum is leptin-independent. Gravitational forces, being proportional to a subject's body mass (*F* = *m* × *g*), have been present throughout evolution. We propose that this information on gravitational force might be used by land-living animals to determine their body weight, avoiding excessive body size that reduces fitness. We, therefore, hypothesized that there might be a homeostatic system that uses gravity for regulation of body weight in land-living species, and we denoted it the *gravitostat* [17]. A possible role of gravitational forces to keep the body weight constant had been proposed already in 1950 by the former Vice President of the Royal Society Charles Dodds but no experimental study supporting this hypothesis was presented by him [18]. The first experimental studies supporting an effect of gravitational forces on body weight regulation used centrifugation to induce hypergravity. A large majority of studies (including an extensive meta-analysis of 18 independent studies), but not all studies, demonstrated that hypergravity induced by centrifugation reduces body mass in rodents [19–24]. In addition, approximately 20 years ago, two independent research groups demonstrated that intraperitoneal implantation of inert weight capsules partly reduced (by *ca* 50% of the added weight) the biological body weight in proportion to the added weights in male but not female rodents on normal chow diet [25–27]. Importantly, we have demonstrated that the homeostatic body mass reducing effect of increased loading by weight capsules is much more efficient in obese mice on high fat diet than in lean mice on normal chow diet [17,28,29]. This key finding explains the previously observed less pronounced effects of increased loading using rodents on normal chow diet as seen by others and initially also by us [17,25–29]. The use of obese mice on high fat diet, mimicking human obesity, enabled us, in several articles, to demonstrate that the loading effect is observed for both male and female mice and for both intraperitoneal and subcutaneous implantation of weight capsules [17,28,29]. Thus, data from three independent laboratories using implantation of weight capsules and multiple independent studies using centrifugation support the notion that increased loading reduces body mass in rodents.

**Figure 2.**
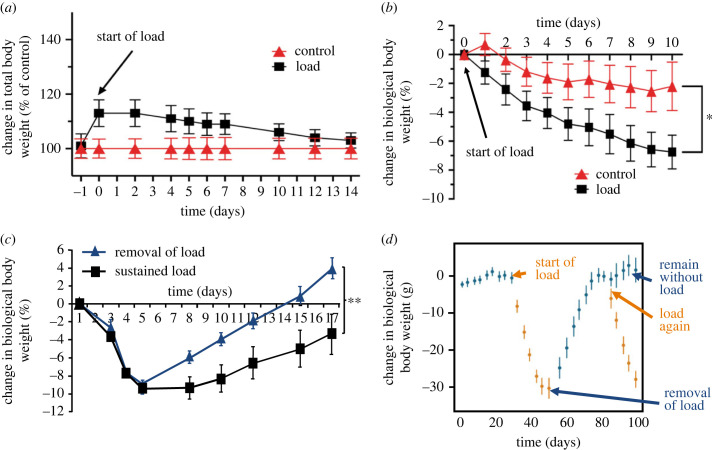
Increased loading decreases body weight in rodents and pigeons. (*a*) Total weight (loading + biological body weight) of mice with intraperitoneal load is normalized after two weeks. Adapted from [17]. (*b*) Biological body weight of mice is decreased to compensate for subcutaneous experimental load. Adapted from [17]. (*c*) Decrease in biological body weight caused by intraperitoneal load is reversible in mice. Adapted from [17]. (*d*) Biological body weight of pigeons is reversibly decreased to compensate for load glued to the back. Adapted from [30].

To experimentally test the gravitostat hypothesis, we used increased loading achieved via either intraperitoneal or subcutaneous implantation of heavy capsules compared with light capsules [17]. We found clear evidence that increased loading in rodents reduced the biological body weight until a new steady state was reached (figure 2*a,b*). These data demonstrate the existence of a compensatory mechanism for the regulation of body weight in rodents with artificially increased loading.

An interesting recent study in homing pigeons indicates that there could a be a gravity-dependent compensatory regulation of body weight also in birds [30]. While investigating the possible adverse effects of bio-loggers adapted to pigeons, Portugal & White found that a 5% increase in load glued to the back of homing pigeons resulted in a compensatory 5% decrease in biological body weight. Removal of the load demonstrated that the effect was reversible in rodents and pigeon (figure 2*c,d*) [17,30]. There are probably many differences in the regulation of body weight between mammals and birds. However, the data on body weight regulation in pigeons (figure 2*d*) indicate that there is homeostatic regulation of body weight based on gravity in animals as divergent as birds and land-living rodents.

We propose that there might be two components of the gravity-dependent regulation of body weight (gravitostat). First, we posit a mechanism involving increased energy consumption in relation to body weight when working against gravity on land, and we propose that this mechanism may be sensor-independent. The laws of physics tell us that force (*F*) must increase when body mass (*m*) increases as the gravitational force increases proportionally to the body mass, while the acceleration due to gravity (*g*) is constant (Δ*F* = Δ*m* × *g*). This is because a heavier individual will work against a larger gravitational force when moving on land and maybe even more so when actively flying through the air. For potential energy, the extra energy required by increased body mass is easy to calculate (ΔEnergy = Δ*m* × *g* × *h*) where *h* is height.

**Figure 3.**
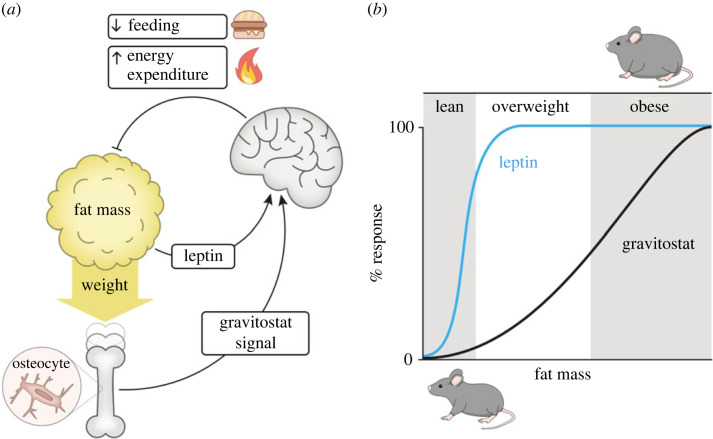
Hypothesis for how leptin and the gravitostat act in concert to regulate body weight homeostasis. (*a*) The leptin and gravitostat systems in the regulation of body weight (adapted from [17]). (*b*) The dose–response curve for the effect of leptin (blue line) is shifted to the left, reflecting that treatment with this hormone decreases body weight mainly in lean animals. By contrast, the dose–response curve for the effect of the gravitostat (black line) is shifted to the right, reflecting that the effect of the gravitostat decreases body weight more efficiently in obese mice. Adapted from [29].

Beside the rather obvious, but often neglected, mechanism discussed above, we propose that there also might exist a specific sensor-dependent component. This proposed component involves sensing of the body weight by osteocytes in the weight-bearing bones, resulting in a feed-back regulation of global energy metabolism and body weight ([17], figure 3*a*). The effect of the gravity-dependent mechanism aims to keep body weight constant (or within a certain interval as described below) by regulating food intake and/or energy burning. Deviations in body weight will be registered by a sensor that signals to an integrating centre, which in turn regulates an effector. This effector in turn causes the partial or total reversal of the body weight.

## 4. Comparison of leptin and gravity-dependent effects on body weight homeostasis

When initially comparing the effects of increased loading and leptin [9], we found evidence that both systems regulate body weight. However, this may be done in different ways.

**Figure 4.**
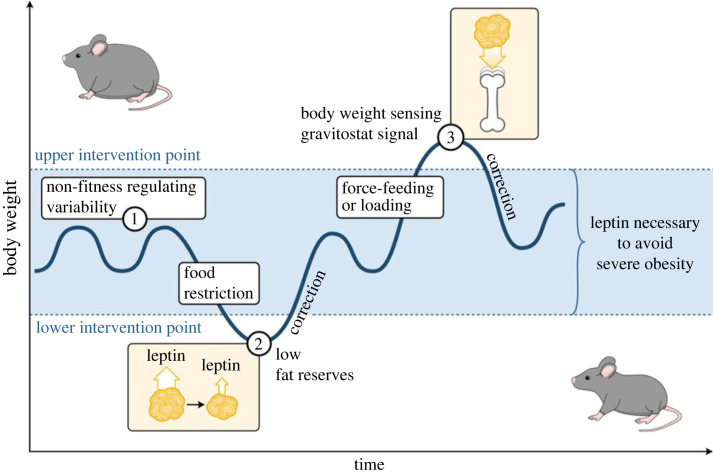
The dual hypothesis of body weight regulation, including gravity-dependent and leptin-dependent actions. For explanation of this figure, please see the main text. Adapted from [16].

For leptin, it is assumed that variations in fat mass (i.e. the size of the fat cells and/or the number of fat cells [31]) are accompanied by similar changes in production and release of leptin. Leptin then suppresses food intake and increases energy expenditure via effects on the brain (figure 4*a*). In particular, leptin seems to stimulate receptors in the satiety centre of the hypothalamus and thereby decrease feeding as well as increase fat burning [11,12].

Regarding the sensor-dependent effect of the gavitostat, we postulate that increased fat mass increases axial pressure on lower extremities (figure 3*a*). Mice with osteocyte depletion displayed attenuated response to increased loading [17] (figure 3*a*). We therefore hypothesize that the effect of increased loading is sensed by the osteocytes, which in turn generate an endocrine and/or nervous signal that acts on the brain to decrease feeding and/or increase fat burning (figure 3*a*). Given that both the leptin system and the sensor-dependent part of the gravitostat are proposed to act on the brain, it may be speculated that they act on converging pathways in the brain and that they provide complementary information about the metabolic condition of the body to an integrating centre [32].

## 5. The leptin system is efficient in lean while the gravity-dependent system is efficient in obese mice

Early on in our studies of the role of gravity-dependent mechanisms for regulation of body weight, we found by serendipity that increased loading seemed to suppress body weight and fat mass more effectively in obese mice. As mentioned above, leptin has been reported to be more effective in lean rodents [29,33]. Comparison of effects seen using different experimental settings can be very misleading. Therefore, in the same study we directly compared the effects of leptin treatment and increased loading on body weight and body fat mass in lean mice and mice with diet-induced obesity [29]. The results clearly showed that there are fundamental differences between the effects of the gravity-dependent mechanism and the effect of the leptin system (figure 3*b*). Leptin treatment decreased body weight in lean but not in obese mice. By contrast, increased loading decreased body weight and body fat more efficiently in obese than in lean mice (figure 3*b*).

## 6. The dual hypothesis of body weight regulation, including gravity-dependent and leptin-dependent actions

John Speakman has suggested that body weight, within an upper and a lower intervention point, is not tightly regulated but may vary (figure 4). The reason for this may simply be that fitness (= the ability to survive to reproductive age, find a mate, and produce offspring) is affected only to a small degree as long as the body weight is kept between the subjects' upper and lower intervention points [1,2]. One reason why fitness in humans has not been affected by moderate variations in body weight could be that humans/hominin ancestors to humans have not been threatened to any large degree by predators during the last 2 Myr [1]. This could be thanks to social collaboration in defence, mastering of fire, development of sharp weapons, the capacity to throw objects etc. [1]. Despite the arguments put forward above, there should logically be a limit to how obese an individual can become, for instance due to a loss of agility. This has been called the upper intervention point by Speakman, but it is completely unknown how body weight is kept under this level [16]. In a wider perspective, there has been little information about the physiological factors that counteract severe obesity, as acknowledged by several authors [2,7,16,34,35]. Regarding the lower intervention point there is more information. It is generally accepted that there is a physiological response to fasting and that this involves low fat levels which in turn cause decreased serum leptin levels. The low leptin levels will then contribute to physiological starvation response, including intense hunger and energy ransoming achieved by turning off several body functions [7].

Based on the fact that body weight is tightly regulated when outside the normal range promoting fitness, either passing below the lower intervention point or above the upper intervention point, we, herein, propose *the dual hypothesis of body weight regulation*, including gravity-dependent and leptin-dependent actions. The dual hypothesis of body weight regulation is based on the concept from Speakman's dual intervention point model but with the addition that the dual hypothesis of body weight regulation also includes the main proposed regulators of body weight both when passing below the lower intervention point (leptin) and when passing above the upper intervention point (gravity-dependent actions; figure 4). According to this hypothesis, when the animals are within the normal range of fat mass between the upper and lower intervention points, no homeostatic body regulation mechanism is active. Then, fitness is only marginally affected by body weight changes (figure 4, point 1). Fasting or food restriction that continues for a prolonged period of time may decrease body weight and fat mass below the lower intervention point, i.e. to a level threatening survival. This decreases leptin release to the bloodstream. The ensuing low leptin levels trigger a response aimed at increasing the chances of survival of the individual, i.e. its fitness. Firstly, intense hunger and eager food seeking may pay off when an individual finds food. Secondly, fasting-induced decrease in fat mass and serum leptin causes rationing of energy, by decreased energy expenditure [7]. Thus, an individual with decreased body weight may correct this and get over the lower intervention point again (figure 4, point 2). It is well established that forced overfeeding results in increased body weight, which is rapidly corrected when the overfeeding stops [16,35]. We hypothesize that the compensatory decrease in body weight is caused by an activation of the gravity-dependent regulation of body weight when the body weight passes above the upper intervention point. Thus, one might speculate that the proposed gravitostat could be involved in the initiation of the unknown catabolic signal in the overfed state, sought after by several authors [16,35,36]. Experimentally, this upper intervention point is passed when obese mice are subject to increased loading (figure 4, point 3).

We acknowledge that the the 'dual hypothesis of homeostatic body weight regulation, including gravity-dependent and leptin-dependent actions’ still only is a hypothesis supported by some evidence described in this Opinion piece. Alternative interpretations, challenging our hypothesis, are that leptin is an important regulator of body weight not only at the lower end of the body weight spectrum but also at the upper end of the body weight spectrum, and that increased loading by implanted capsules or centrifugation may reduce body weight also via an increased stress response [21,37]. Therefore, further studies are warranted to determine if leptin and gravity-dependent mechanisms act together to maintain body weight within normal range.

## 7. Conclusion

Body weight is tightly regulated when outside the normal range promoting fitness. We hypothesize that leptin and gravity-dependent mechanisms act together to maintain body weight within normal range, promoting fitness. We, herein, propose the *dual hypothesis of body weight regulation*, including gravity-dependent actions at the upper end and leptin-dependent actions at the lower end of the body weight spectrum.

## Data Availability

We have permission to reproduce data from [17].
